# Experimental study on variation law of electrical parameters and temperature rise effect of coal under DC electric field

**DOI:** 10.1038/s41598-021-86598-0

**Published:** 2021-03-30

**Authors:** Yunpeng Yang, Zhihui Wen, Leilei Si, Xiangyu Xu

**Affiliations:** 1 State Key Laboratory Cultivation Base for Gas Geology and Gas Control, Henan Polytechnic University, Jiaozuo, 454000 China; 2 College of Safety Science and Engineering, Henan Polytechnic University, Jiaozuo, 454003 China; 3 State Collaborative Innovation Center of Coal Work Safety and Clean-Efficiency Utilization, Jiaozuo, 454003 China

**Keywords:** Mineralogy, Energy science and technology, Atomic and molecular physics

## Abstract

Joule heats which are generated by coals in an applied electric field are directly correlated with variation resistivity of electrical parameters of coals. Moreover, the joule heating effect is closely related with microstructural changes and relevant products of coal surface. In the present study, a self-developed applied direct current (DC) field was applied onto an experimental system of coals to investigate variation resistivity of electrical parameters of highly, moderately and lowly metamorphic coal samples. Moreover, breakdown voltages and breakdown field intensities of above three coal samples with different metamorphic grades were tested and calculated. Variation resistivity of electrical parameters of these three coal samples in 2 kV and 4 kV DC fields were analyzed. Results show that internal current of all coal samples increases continuously and tends to be stable gradually after reaching the “inflection point” at peak. The relationship between temperature rise effect on anthracite coal surface in an applied DC field and electrical parameters was discussed. The temperature rise process on anthracite coal surface is composed of three stages, namely, slowly warming, rapid warming and slow cooling to stabilize. The temperature rise effect on anthracite coal surface lags behind changes of currents which run through coal samples. There’s uneven temperature distribution on anthracite coal surface, which is attributed to the heterogeneity of coal samples. In the experiment, the highest temperature on anthracite coal surface 65.8 ℃ is far belower than the lowest temperature for pyrolysis-induced gas production of coals 200 ℃. This study lays foundations to study microstructural changes and relevant products on coal surface in an applied DC field.

## Introduction

Most of coal seams in China are characteristic of high gas content and low permeability, which influence gas extraction efficiency significantly^1, 2^. Many experts and scholars have proposed technologies to strengthen gas extraction from coal seams. Mechanical technologies mainly include high-pressure hydraulic measures, explosion-induced cracking, gas injection displacement of coal seams, etc^3–6^. Technologies that apply applied physical field mainly focus on changing adsorptivity of coal seams by using alternative electromagnetic field^7–11^ and alternative electric field^12, 13^. Recently, researches on effects of applied DC field on gas adsorption characteristics of coals and permeability improvement of cracked coals by electric heats have achieved new progresses. Li et al.^14^ carried out an experimental study on gas adsorptivity of coals under the action of a DC field, and found that voltage can weaken gas adsorption capacity of coals. Zhan^15^ tested and analyzed coals’ isothermal adsorption characteristics of gases under the combined action of DC field and AC field, finding that the DC-AC combined fields could decrease gas adsorption capacity of coals more significantly compared to single field. Fazhi and Boquan et al. carried out a breakdown test of coals by constructing a high-pressure electrothermal cracking coal test system^16–18^. According to experimental results, a lot of pores and cracks are formed in the electrothermal cracking coals and there were violent “acoustic and optical” phenomena in the breakdown process of coals, accompanied with generation of scorching gases.

The process of applying an electric field onto coals is accompanied with joule heating effect. Xu et al.^19^ discussed influences of joule heating effect on electrical conductivity of coals based on a case study of anthracite coal and pointed out that coal temperature increased due to joule heating effect and electrical conductivity of coals was enhanced by increasing currents in coals. Zhu Bo carried out a theoretical study on critical value of gas adsorption capacity in a constant electric field. He found that the final gas adsorption capacity coals was determined by the mutual competition between the temperature rise caused by joule heating effect and the deepening of adsorption well on coal surface as a response to the applied electric field^20^. Zhang Li et al. found through an associated study that the application of an alternating electromagnetic field might trigger loss of dielectric in coals and coal temperature increased accordingly, thus strengthening desorption and diffusion capacities of gases^21^^.^

Both mechanical measures and application of applied electric field change physical mechanics and surface properties of coals, and even generate new micromolecular gases under mechanochemistry^22–24^. Previous studies ignore the joule heating effect of applied electric field on microstructure and properties of coal surfaces. In particular, rare studies have discussed variation resistivity of electrical parameters of coal samples and temperature rise effect on coal surface under the action of an applied DC field. In this study, breakdown field intensity of testing coal samples was analyzed and calculated, variation resistivity of electrical parameters and surface temperature of coal samples under long-term loading of an applied DC field were tested. The purpose is to ensure that the internal structure of coal remains intact when it is lower than the breakdown voltage, which is conducive to temperature testing and the study of chemical changes in the microstructure of the coal surface. Research conclusions provide theoretical references to study crack-induced permeability improvement and evolutions of surface properties of coal samples under the action of applied DC field.

## Experimental system and programs

### Experimental system and principle

**Figure 1:**
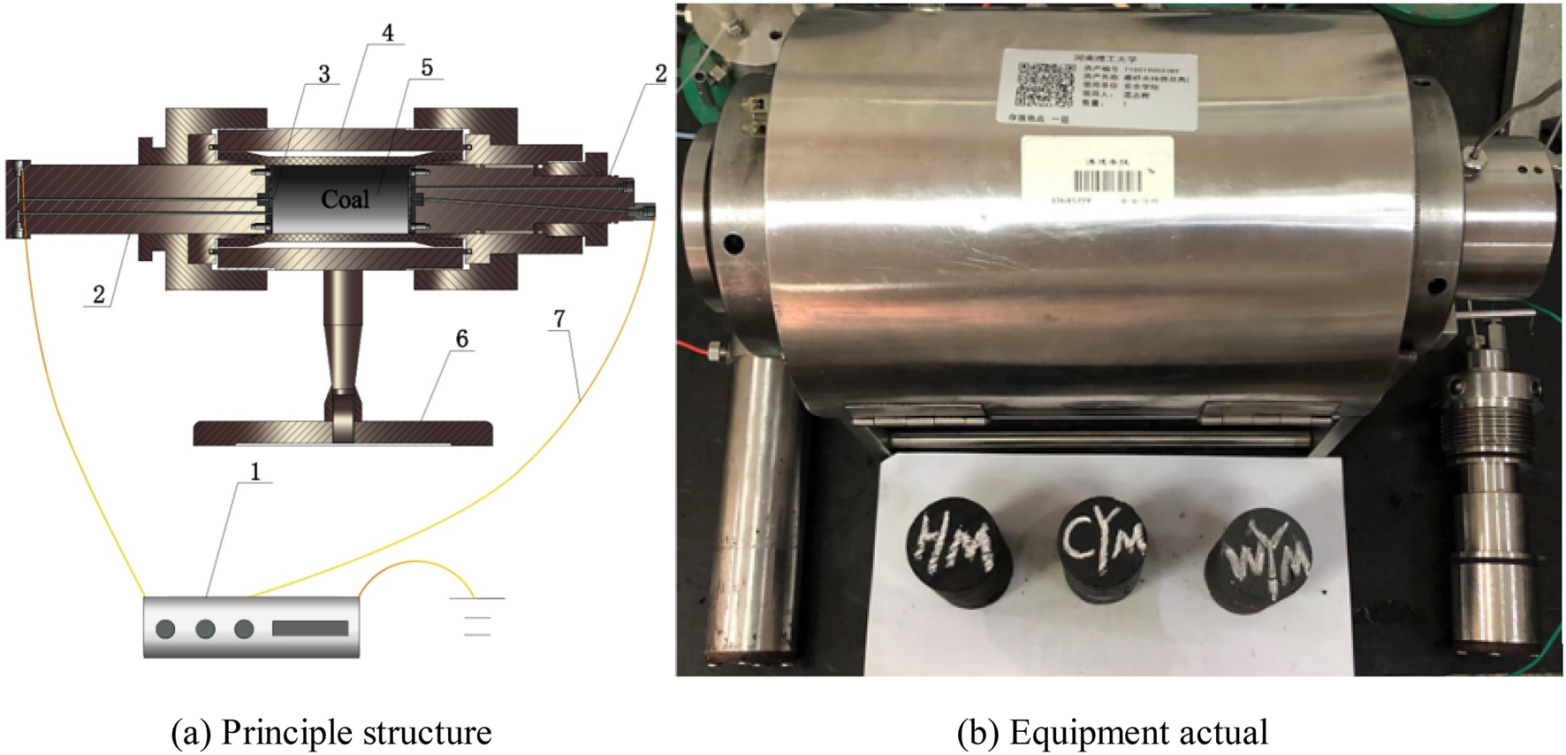
Structure of experimental apparatus for coal sample testing in an applied DC field. 1—high voltage power supply; 2—rod electrodes; 3—red copper electrode plates; 4—holder; 5—coal sample; 6—insulating support; 7—power line.

The loading test system for coals in an applied DC field is mainly composed of a high voltage power supply, a holder, rod electrodes and red copper electrode plates (Fig. 1). The working principle of this system is introduced as follows. Two rod electrodes fix coal samples at the holder. There are bakelite gaskets at the ends of each electrode which extends into the chamber of the holder. Round grooves are set at the centers of two bakelite gaskets, in which red copper electrode plates are embedded. Red copper electrode plates are connected to the positive and negative anodes of the high voltage power supply through power lines. Coal samples can form tight fitting with electrodes in the holder. The TRC2025P high voltage power supply was applied to provide 220 V/50 Hz alternating currents (AC) and the maximum output DC voltage was 12 kV. The whole experimental system was grounded according to national standards, with a ground resistance of lower than 4Ω.

### Testing samples and preparation

Lignite from the Erdos Dongsheng Coalfield (No.: HM, low metamorphic grade), long-flame coal from Gengcun Mine of Yima Coalfield (No.: CYM, moderate metamorphic grade) and anthracite coal from Guhanshan Mine in Jiaozuo Coalfield (No.: WYM, high metamorphic grade) were chosen as testing samples. All coal samples were transported to the laboratory and prepared into 100.00 mm high cylinder samples with a diameter of 50.00 mm. Each group had 12 cylinder samples. All coal samples were dried in a vacuum drying oven for 48 h under 60 °C to eliminate moisture content. Subsequently, testing coal samples were treated by vacuum degassing for 48 h to eliminate the small amount of residual adsorbed gases. Finally, coal samples were sealed up in a drying oven for later test.

### Experimental scheme and steps

According to researches of associated scholars^16, 19, 21^, electrical parameters and temperature of coal samples influence mutually under the action of an applied DC field. In this experiment design, electrical parameters and temperature of coal samples were tested and analyzed, respectively.

#### Test of electrical parameters of coal samples

1. Breakdown voltage test of coal samples
  Coal samples were put into the holder and fixed by loading axial pressure with 0.3 MPa and confining pressure with 0.5 MPa, so that the coal samples were kept stable with the electrode during the electric field loading process. The high voltage power supply was turned on and the output voltage was preset at 1.0 kV. If the coal samples failed to breakdown under the preset loading pressure, the loading voltage was increased at an interval of 0.1 kV until breakdown. Breakdown voltage tests were repeated by 3 times for coal samples at three metamorphic grades, respectively. The critical breakdown field intensities of coal samples were calculated according to test results of breakdown voltage.
2. Variations of currents in coal samples with time

According to above test results, loading voltages were set 2 kV and 4 kV for long-term DC loading test of coal samples with different metamorphic grades. Steady currents of coal samples were measured every 5 min and steady currents in at least continuous 60 min were measured to analyze variation resistivity of currents in coal samples with loading time.

#### Test of temperature rise effect on coal surface

Thermal infrared imager determines temperature on object surfaces through infrared radiation energy from the object rather than through direct contact. Since metal cylinders can influence infrared radiation energy during temperature rise of coal samples, coal samples were loaded with voltage in an external environment to test temperature. Output DC voltage (lower than the breakdown voltage) from the high voltage power supply was set for loading of coal samples. Surface temperatures on coal samples at different loading stages were tested with an YRH600 thermal infrared imager. Cloud maps of infrared temperature distribution on coal sample surface were captured every 5 min, totally for at least continuous 90 min. The maximum temperature on coal surface and temperature distribution characteristics were gained using the SatIrWizard software to analyze variation resistivity of surface temperature of coal samples. Parameters of thermal infrared imager were set as follows: temperature testing distance = 1 m, radiance = 0.95, ambient temperature = 24.0 ℃, relative humidity = 80% and temperature correction = 0.0 °C.

## Experimental results and analysis

### Tests and analysis of breakdown voltage and breakdown field intensity

Breakdown field intensity is a physical variable that reflects electric strength of dielectric substances. There are three breakdown forms for solid dielectrics^25–27^:

1. Electric breakdown is the phenomenon that electric field makes dielectric lose the insulting properties by accumulating enough quantity and energy of charged particles in it.
2. Thermal breakdown means that dielectric loses insulating properties upon excessive temperature caused by accumulation of heats in an electric field.
3. Electrochemical breakdown means that dielectric develops chemical changes slowly as a response to the collaborative effects of electric field and temperature, which result in gradual deterioration of performances and finally losing insulation properties.

During breakdown voltage test of coal samples, there’s no heat accumulation due to the relatively low preset-voltage, continuous and adjustable growth rate of voltage, and short experiment period. Therefore, electric breakdown of coal samples took the dominant role in the experiment. Since the copper electrode plate in the holder is not completely attached to the coal sample, the distance between the two electrodes after measurement was 101.00 mm. Three breakdown experiments were performed to coal samples of high, moderate and low metamorphic grades, respectively. The electric field intensity between electrodes can be calculated as Eq. (1):

1 $$E = \frac{U}{d}$$

where E is the electric field intensity between electrodes (kV/cm), U is voltage between electrodes (kV), and d is the space between two electrodes (cm).

Test results of breakdown voltage and breakdown field intensity of different coal samples are listed in Table 1.

**Table.1:** Test results of breakdown voltages and field Intensities of different coal samples.

Coal type	Sample no	C_adf_ %	Break down voltage kV	Breakdown field intensity kV/cm	Resistivity Ω m
Lignite	HM-1	57.021	9.3	0.920	5763.15
	HM-2	58.436	8.5	0.841	5689.49
	HM-3	59.087	7.9	0.782	5417.23
Long-flame coal	CYM-1	74.035	7.5	0.724	3928.35
	CYM-2	75.213	6.4	0.633	3727.14
	CYM-3	77.852	7.6	0.752	4192.12
Anthracite coal	WYM-1	87.265	5.0	0.495	3435.17
	WYM-2	90.714	5.7	0.564	3624.22
	WYM-3	89.532	4.2	0.416	3237.54

**Figure 2:**
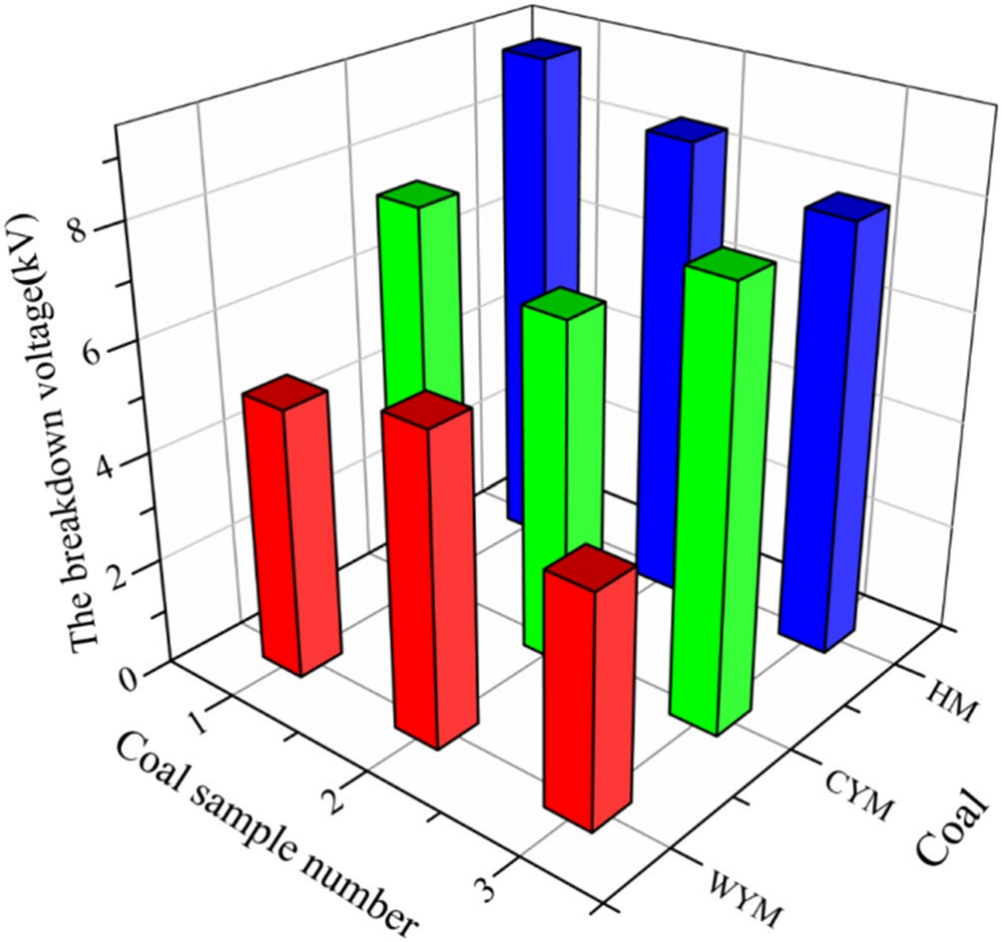
Breakdown voltage distribution among three coal samples at different metamorphic grades.

It can be seen from Table 1 that the higher the resistivity between the same coal samples and the smaller the fixed carbon content, the higher the breakdown voltage. The resistivity of different coal samples decreases with the increase of metamorphic degree. In addition under the same experimental conditions, breakdown voltage and field intensity differ significantly among different coal samples even though the coal size is identical. Breakdown field intensity is determined by breakdown voltage. The distribution pattern of breakdown voltage was drawn to reflect differences of breakdown voltage among different coal samples clearly and intuitively (Fig. 2).

According to tests and analysis, coal samples at different metamorphic grades show significantly different breakdown voltage and breakdown field intensity. This is because coals are inhomogeneous media with dual pore characteristics and there’s uneven distribution of mineral in coals^28^. The primary cracks and mineral distribution in solid media can affect breakdown voltage significantly^29^. Therefore, breakdown voltage of the same coal type with same size might vary to some extent. The average breakdown voltage and breakdown field intensity of lowly metamorphic coals are higher compared to those of highly metamorphic coals. Reasons can be explained as follows. With the increasing degree of coalification, carbon content in coals increases and electrical conductivity of coals might soar up continuously when the carbon content increases to 92%^30^, finally resulting in the breakdown of highly metamorphic coals under a relatively low voltage.

At the same time, the coal conductivity is not only related to the degree of coal metamorphism, but also closely related to the internal temperature of the coal^31–33^. Relevant scholars have studied the law of coal body resistivity changing with temperature, and found that when the coal body temperature is between 30 and 90 ℃, the resistivity decreases linearly with the increase of temperature. The voltages 2 kV and 4 kV applied in this paper are both below the breakdown voltage of the coal body. The purpose is to test the electrical parameters and surface temperature rise based on the coal body structure without damage.

In this work, we used infrared thermal imager to test the surface temperature change of coal. Under such condition, we assumed that the local temperature of any carbon–carbon connection between the particles in the sample will not lead to any chemical reaction (and additional carbonization) that changes the internal carbon structure.

### Variation laws of currents in coals under the action of an applied DC field

Coal is a typical dielectric material. According to types of electrical conductivity, coals can be divided into electron conduction type, ionic conduction type, electron-ionic mixed conduction type and hole conduction:

1. Electron conduction is based on a certain amount of free radicals in molecular structure of coals. These free radicals gain energies from the applied electric field to get rid of original binding and jump along the electric field, forming electron flows.
2. Ionic conduction is mainly attributed to the high contents of water and mineral in coals. These mineral contain ionic compounds which can form positively charged ions that can move freely under the action of water, thus forming currents along the electric field.
3. Mixed conduction means that dielectric conduction contains both ionic conduction and electron conduction. These three conduction types often coexist in coals, with differences in the dominant conduction type.
4. Hole conduction means that some valence electrons of covalent bonds in coal gain some energy due to thermal movement, and thus get rid of the constraints of covalent bonds and become free electrons. At the same time, holes are left on the covalent bonds, and the holes have net surplus. The positive charge will attract other electrons around to fill the holes, and then form holes to move in the crystal to conduct electricity^34^.

**Figure 3:**
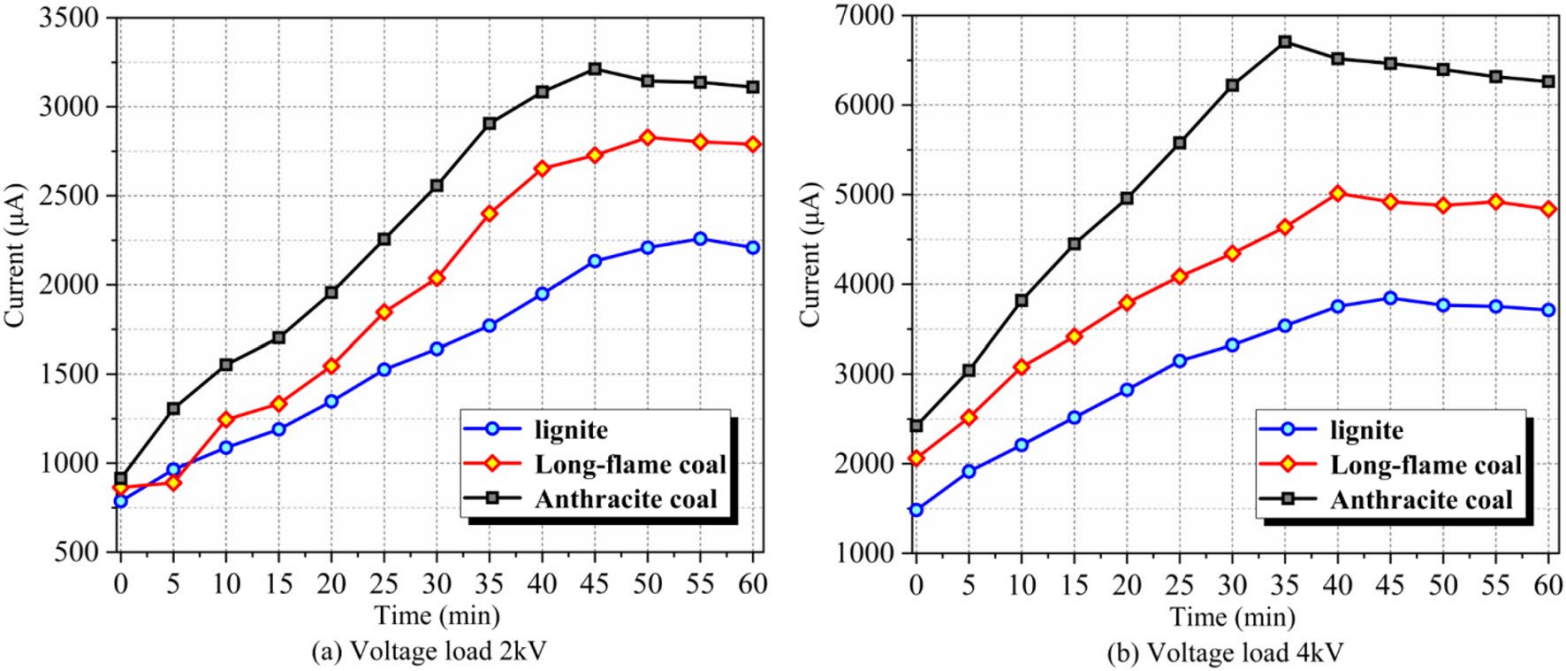
I–t curves of different coal samples.

Coal samples in this experiment were processed by vacuum drying and degassing before current test to eliminate water and gas. As a result, electron conduction took the dominant role in coal samples. According to test and analysis, changes of currents in different coal samples with time in the applied DC field were disclosed, which demonstrated and verified which demonstrated and verified variation laws of electrical parameters of coal samples (Fig. 3).

Coal is a unique dielectric material and free electrons in coals change from scattering to directed arrangement under the action of applied DC field, forming moving current beams. With the increase of voltage, these electrons are easier to get rid of binding from groups and thereby form more electrons in the free excited state, thus increasing the current continuously. Therefore, it can be seen from the I–t curve in Fig. 3 that given the same loading voltage, currents in all coal samples increase significantly with the increase of the conduction time. Specifically, when the loading voltage is 2 kV, currents in lignite increases to the maximum 2259 μA from the initial 787 μA. Currents in long-flame coal increase from the initial 864 μA to the maximum 2827 μA. Moreover, currents in anthracite coal increases from the initial 913 μA to maximum 3212 μA. When the loading voltage is 4 kV, currents in lignite increases to the maximum 3845 μA from the initial 1483 μA. Currents in long-flame coal increase from the initial 2060 μA to the maximum 5013 μA, and currents in anthracite coal increases from the initial 2422–6704 μA.

The changes of currents which run through coal samples are attributed to changes of resistances, which were caused by temperature affects the connection between local carbon crystallites^35^. Therefore, dynamic variation characteristics of currents in coal samples can reflect variation resistivity electrical parameters of coal samples in applied electric fields indirectly. In this experiment, the growth amplitude and rate of currents in anthracite coal are higher than those of long-flame coal and lignite. This is because there’s small binding strength to electrons in molecular structure of anthracite coal and the possibility of electron transition is increased significantly due to the relatively high ordered aromatic degree^36^.

As the loading test continued, currents in all coal samples reached the “inflection points”. This is related with heat exchange with the external world. When joule heats generated by currents in coal samples are equivalent to the escaping heats to the outside, conduction characteristics of coal samples and currents that run through coal samples became stable. Accordingly, specific resistance of coal samples became basically stable.

In Fig. 3a, lignite, long-flame coal and anthracite coal reach the inflection points at 55 min, 50 min and 45 min when the loading voltage is 2 kV. In Fig. 3b, lignite, long-flame coal and anthracite coal reach the inflection points earlier at 45 min, 40 min and 35 min when the loading voltage is 4 kV, respectively. These reveal that increasing loading voltage of coal samples can make coal samples reach the inflection point of peak current earlier.

### Temperature rise effect on coal surface in an applied DC field

Under the action of an applied DC field, alkane side chains and functional groups with low bond energy on coal surfaces break and fall off, which causes macromolecular structural changes and generate new micromolecular gases on coal surfaces^37–40^. Coals can generate pyrolysis gases like CH_4_, H_2_ and CO when they are heated to 200 ℃ in inert atmosphere^41–43^ Therefore, studying temperature rise effect on coal surface under the action of an applied DC field is the key to distinguish gas production from “electrochemical” effect from gas production from pyrolysis caused by joule heating effect.

Coal is a typical inhomogeneous multiphase medium. When excessive voltages are applied at two ends of coal samples, a plasma channel is formed in coal samples to break coals under the collaborative influences of electric field stress and thermal expansion stress^17^. In this experiment, the loading voltages at two ends of coal samples were maintained below breakdown voltage, which were inadequate to breakdown of coal samples. However, internal and surface temperatures of coal samples will surely increase under the continuous action of an applied DC field due to the joule heating effect. According to the Lenz’s law^12^ and specific heat formula^44^, the relationship between temperature rise and loading voltage of coal samples can be disclosed. The power loss of coal samples under the action of an applied DC field is:

2 $$P = \frac{U}{R}^{2}$$

The adsorbed heats of coal samples are:

3 $$Q = kP$$

The temperature rise quantity of coal samples is:

4 $$\Delta T = \frac{Q}{mc} = k\frac{{U^{2} }}{Rmc}$$

where U is the applied voltage (kV), R is the equivalent resistance of coal samples (Ω), c is specific heat capacity of coal samples (J/kg °C), m is mass (kg, and k is the coefficient of heats transformed from power loss and it is dimensionless.

**Figure 4:**
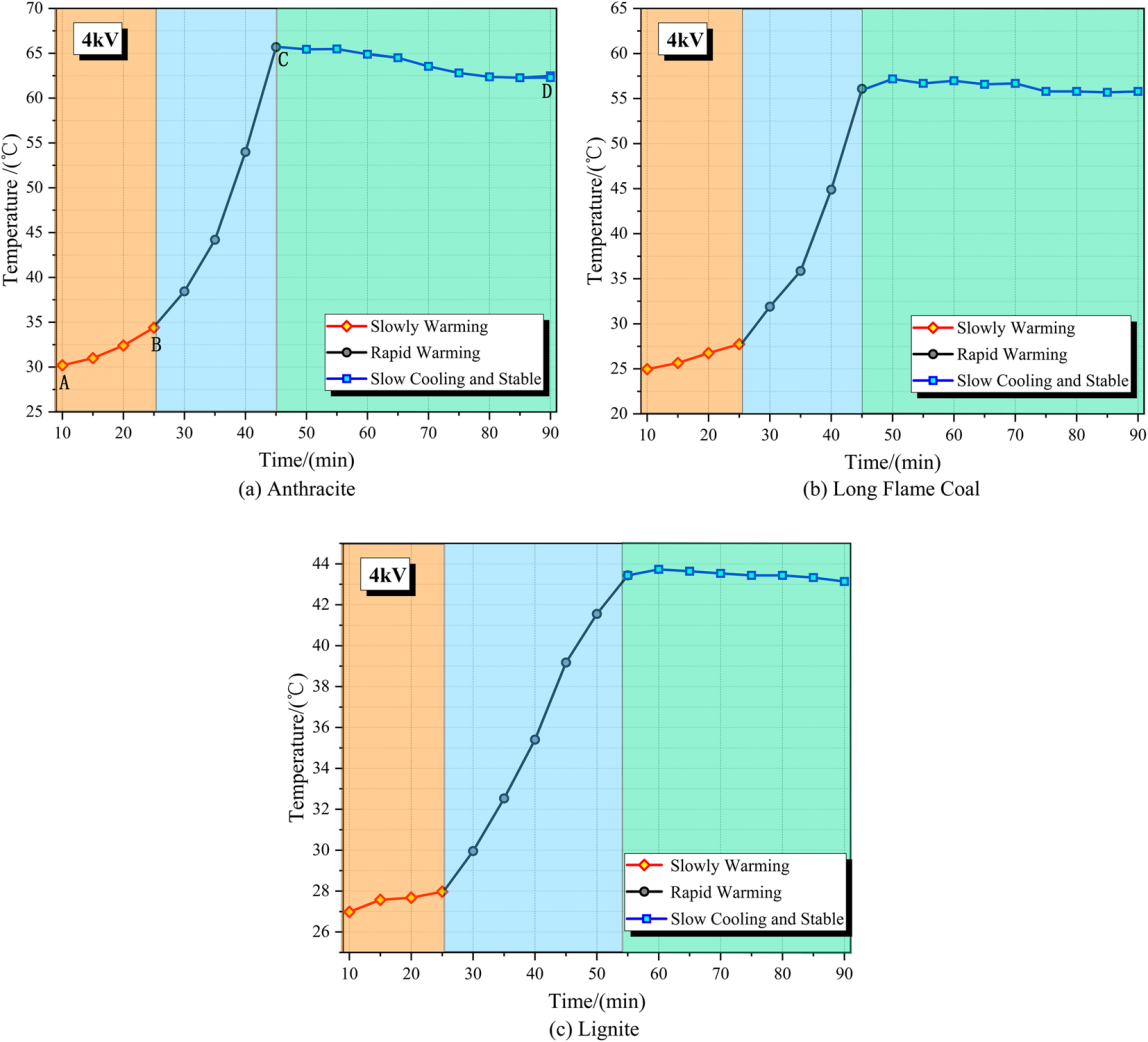
Variation curve of surface temperature of coal with different metamorphic degree under 4 kV.

According to test and analysis in “Variation laws of currents in coals under the action of an applied DC field” section, anthracite coal shows the highest growth rate of current and amplitude of peak current under the same loading voltage. Therefore, anthracite was chosen to analyze temperature rise effect on coal surface in an applied DC field in the following text. During the test, temperatures on coal surface were tested every 5 min since a DC field was applied onto the coal sample for 10 min. On this basis, the variation curve of highest temperature on anthracite coal surface with time was drawn (Fig. 4).

In a 4 kV applied DC field, temperature on anthracite coal surface experiences three stages (Fig. 4), which are the slowly warming stage (10–25 min, section AB), rapid warming stage (25–45 min, section BC) and slow cooling stage (45–90 min, section CD).

**Figure 5:**
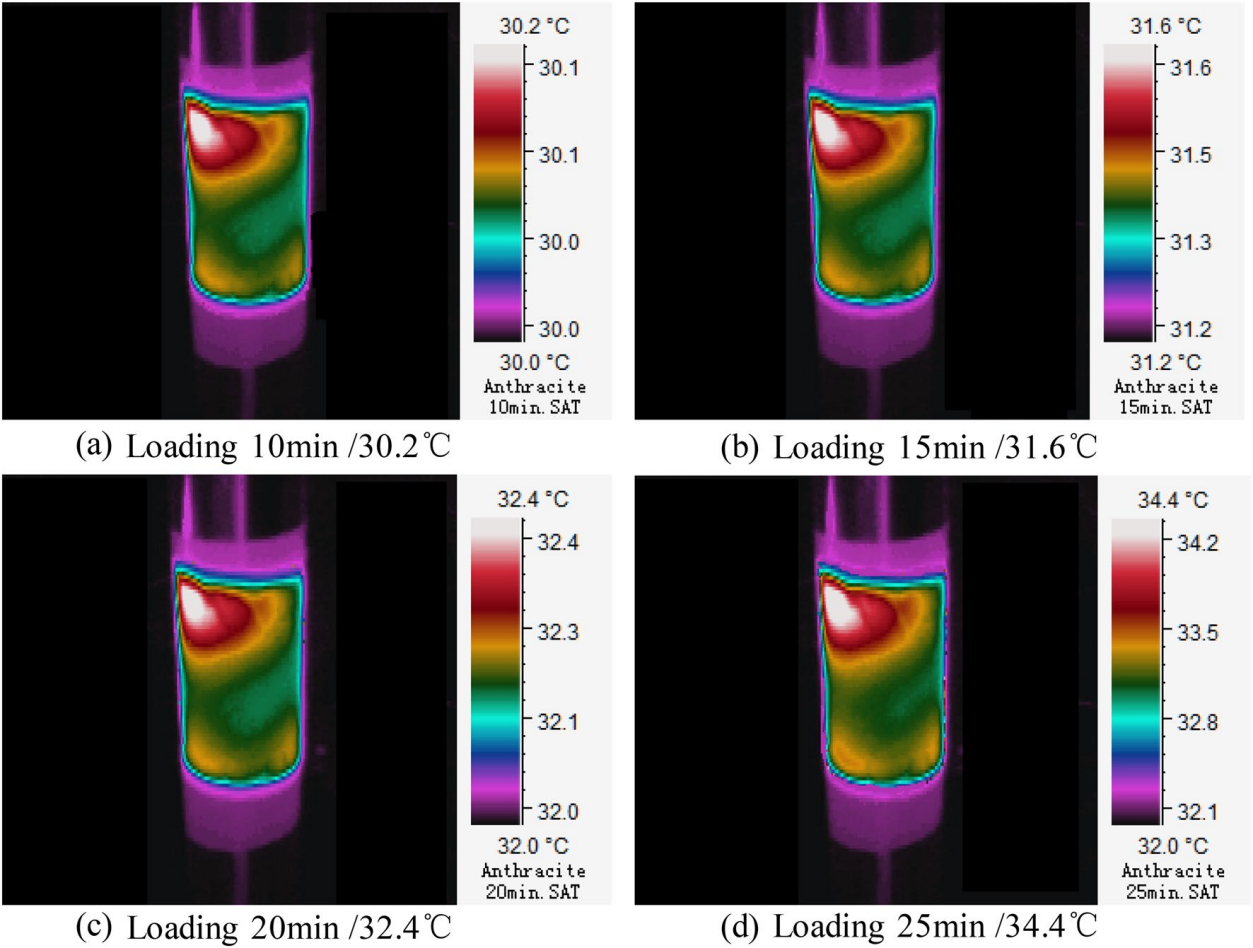
Three-dimensional infrared cloud maps of slowly warming stage.

**Figure 6:**
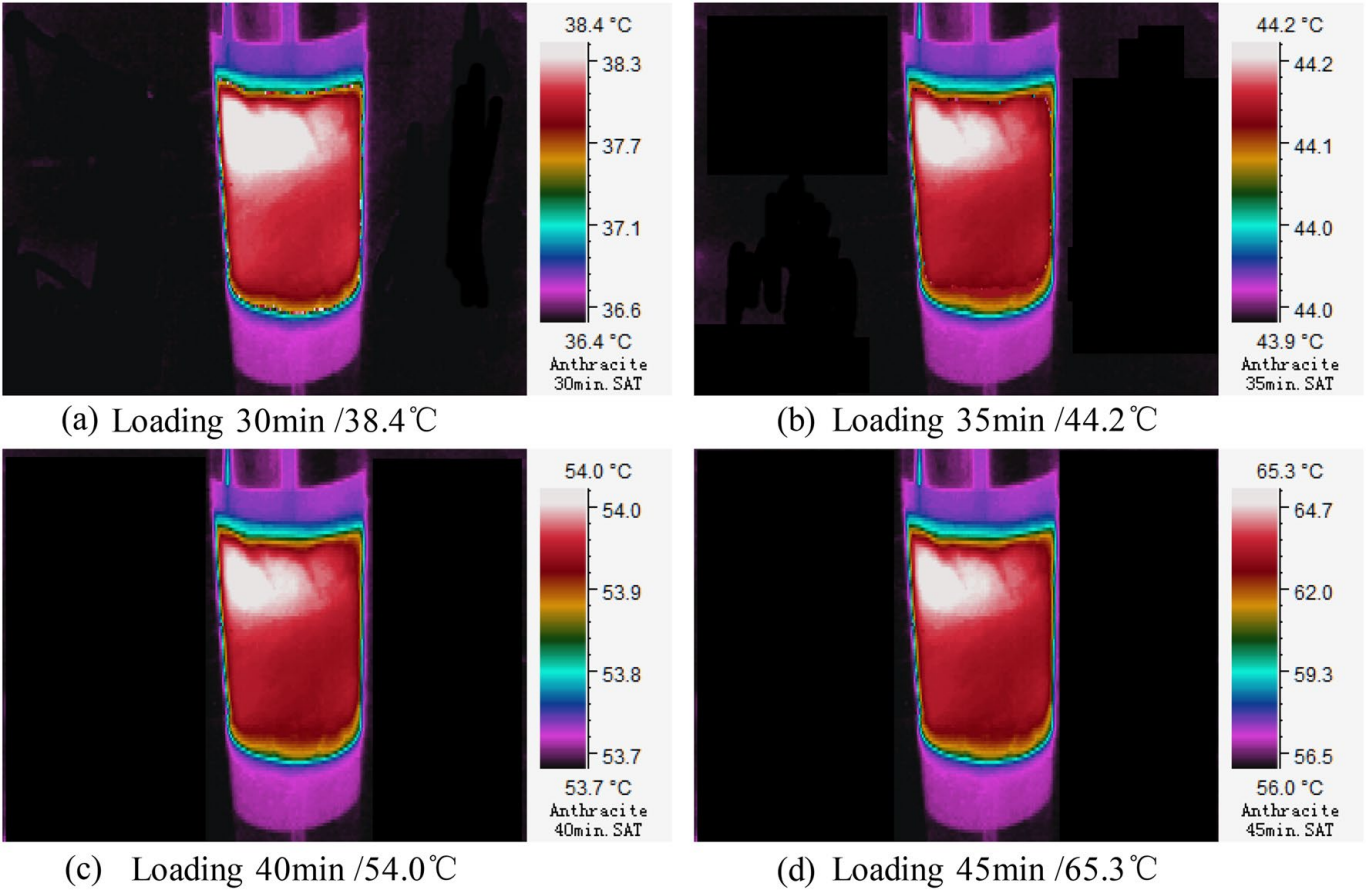
Three-dimensional infrared cloud maps of rapid warming stage.

**Figure.7:**
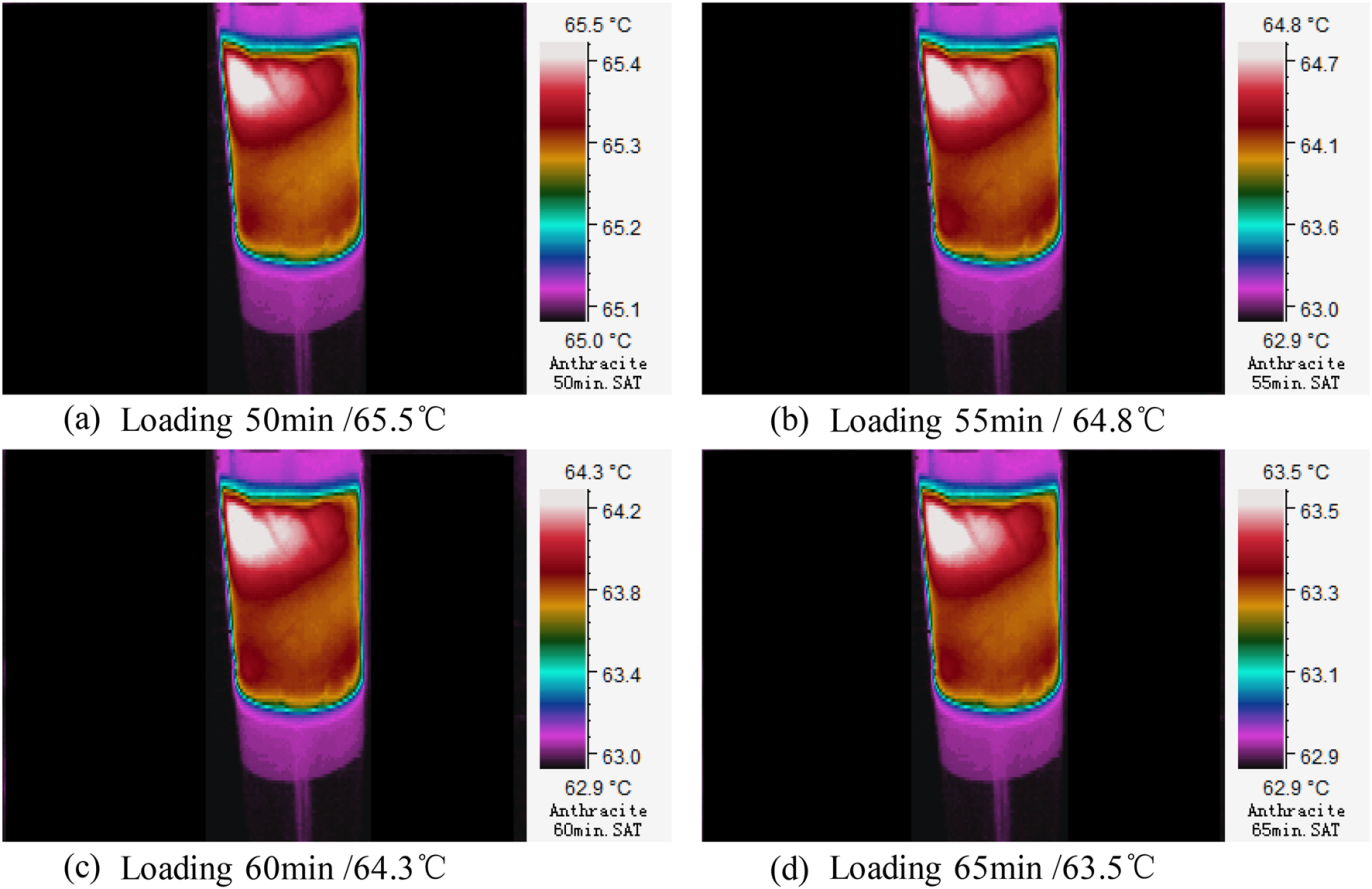
Three-dimensional infrared cloud maps of slow cooling stage.

Temperature rise on coal sample surface is a dynamic process. Moreover, it is found from the experiment that temperature distribution on coal sample surface is uneven. The infrared distribution cloud map of temperatures on coal samples in above three stages were collected (Figs. 5, 6, 7) to characterize and reflect the continuous variation of surface temperature accurately.

1. Slowly warming stage (Section AB)
  It can be seen from Fig. 4 that the highest temperature on anthracite coal surface increases slowly within the section AB (10–25 min) in an applied DC field. In this stage, the highest temperature changes between 30.2–34.4 ℃, the temperature variation is △T = 4.2 ℃, and the average temperature rise rate is T_v_ = 0.28 ℃/min. Distribution patterns of temperature on coal surface and variation laws in the slowly warming stage are shown in Fig. 5. Obviously, there’s uneven distribution of temperatures on coal surface, manifested by local heat accumulations. Meanwhile, it can be concluded from a comparative analysis of Fig. 5a,b that the surface temperature on anthracite coal increases slowly. Coal is a special dielectric similar to an energy storage capacitor. It can store the energy of the electric field in the initial stage of voltage loading, reducing the current at both ends of the load, resulting in a weaker Joule heating effect.
2. Rapid warming stage (Section BC)
  It can be seen from Fig. 4 that slope of the variation curve of highest temperature on anthracite coal surface increases significantly within the section BC (25–45 min) in an applied DC field. In this stage, the highest temperature changes between 38.4–65.3 ℃, the temperature variation is △T = 26.9 ℃, and the average temperature rise rate is T_v_ = 1.79 ℃/min. Similarly, there’s uneven distribution of temperatures on coal surface. Due to accumulation of loading time and joule heats in the experiment, electrical parameters of coal samples are changed and equivalent resistance declines. As a result, currents that run through coal samples increase greatly, thus resulting in the rapid temperature rise on anthracite coal surface.
3. Slow cooling stage (Section CD)

It can be seen from Fig. 4 that in the section CD (45–90 min), the highest temperature on anthracite coal surface reaches the peak at 45 min, then decreases slowly within 55–75 min, and finally becomes stable after 75 min in an applied DC field. In this stage, the highest temperature changes between 65.5–63.5 ℃, the temperature variation is △T =  − 2.0 ℃, and the average temperature rise rate is T_v_ ≈ − 0.13 ℃/min. According to the infrared temperature distribution cloud map on coal sample surface, temperature is also distributed unevenly (Fig. 7). In this stage, temperature on coal sample surface declines slowly and then becomes stable, finally fluctuating at about 63 ℃. At this moment, the joule heats generated by currents that run through coal samples are equivalent to the escaping heats from the experimental system to the external environment, so that temperature on coal sample surface and electrical parameters tend to be stable.

According to a comparative analysis of Figs. 5, 6 and 7, temperature changes on anthracite coal surface in an applied DC field have following characteristics.

1. There’s uneven temperature distribution on coal sample surface. In the temperature rise process, regions with high temperature is close to the upper ends of coals. This is related with the uneven mineral distribution in coal samples and different specific heat capacity of different minerals.
2. According to a comparative study on I-t curves of anthracite coal samples in a 4 kV applied DC field (Fig. 3b), variations of the highest temperature and temperature rise effect on coal sample surface lag behind changes of currents that run through coal samples for 5–10 min.
3. In the process of temperature rise on anthracite coal surface, the highest temperature is lower than 70 ℃, which is far low the lowest temperature (200 ℃) for pyrolysis-based gas production of coals. Therefore, gas production of coals in an applied DC field is not attributed to pyrolysis.

## Conclusions

Based on experimental tests of changes of electrical parameters and surface temperature of coals in an applied DC field, some major conclusions could be drawn through calculation and analysis.

1. Coals at different metamorphic grades show significantly different breakdown voltages and field intensities, which are determined by heterogeneity and internal mineral distribution of coals. Under the same experimental conditions, lignite shows the highest breakdown voltage and field intensity, followed by long-flame coal and anthracite coal successively.
2. Electron conductivity takes the dominant role in dry coal samples. As the loading test in an applied DC field continues, currents that run through coal samples increase significantly. Moreover, anthracite coal with the highest metamorphic grade presents higher growth rate and amplitude of currents compared to coal samples with moderate and low metamorphic grade.
3. Specific resistance of coal samples is mainly determined by its conductivity and temperature. When the joule heats generated by currents that run through coal samples are equivalent to escaping heats to the external world, the conduction characteristics of coal samples and currents that run through coal samples tend to be stable. Currents in coal samples reach peaks and then become stable after the “inflection points”.
4. In an applied DC field, the highest temperature on anthracite coal surface experiences three stages, namely, slowly warming stage, rapid warming stage and slow cooling to stabilize stage. Besides, the temperature rise effect on coal sample surface lags behind changes of currents that run through coal samples.
5. The highest temperature on anthracite coal surface caused by temperature rise effect in an applied DC field is far lower than the lowest temperature (200 ℃) for pyrolysis-based gas production of coals. Research conclusions lay experimental foundations for gas production of coals based on microstructural excitation in an applied DC field.
